# Use of i-scan Endoscopic Image Enhancement Technology in Clinical Practice to Assist in Diagnostic and Therapeutic Endoscopy: A Case Series and Review of the Literature

**DOI:** 10.1155/2012/193570

**Published:** 2012-12-02

**Authors:** Shawn Hancock, Erik Bowman, Jyothiprashanth Prabakaran, Mark Benson, Rashmi Agni, Patrick Pfau, Mark Reichelderfer, Jennifer Weiss, Deepak Gopal

**Affiliations:** ^1^Division of Gastroenterology and Hepatology, School of Medicine and Public Health, University of Wisconsin, 4th Floor, UWMF Centennial Building, 1685 Highland Avenue, Suite 4000, Madison, WI 53705-2281, USA; ^2^Department of Pathology and Laboratory Medicine, School of Medicine and Public Health, University of Wisconsin, Madison, WI 53705, USA

## Abstract

*Background*. i-scan is a software-driven technology that allows modifications of sharpness, hue, and contrast to enhance mucosal imaging. It uses postimage acquisition software with real-time mapping technology embedded in the endoscopic processor. *Aims*. To review applications of i-scan technology in clinical endoscopic practice. *Methods*. This is a case series of 20 consecutive patients who underwent endoscopic procedures where i-scan image enhancement algorithms were applied. The main outcome measures were to compare mucosal lesions with high-definition white light endoscopy (HD-WLE) and i-scan image enhancement for the application of diagnostic sampling and therapy. *Results*. 13 cases involving the upper GI tract and 7 cases of the lower GI tract are included. For upper GI tract pathology i-scan assisted in diagnosis or therapy of Barrett's esophagus with dysplasia, esophageal adenocarcinoma, HSV esophagitis, gastric MALT lymphoma, gastric antral intestinal metaplasia with dysplasia, duodenal follicular lymphoma, and a flat duodenal adenoma. For lower GI tract pathology i-scan assisted in diagnosis or therapy of right-sided serrated adenomas, flat tubular adenoma, rectal adenocarcinoma, anal squamous cell cancer, solitary rectal ulcer, and radiation proctitis. *Conclusions*. i-scan imaging provides detailed topography of mucosal surfaces and delineates lesion edges, which can directly impact endoscopic management.

## 1. Introduction

As the field of gastrointestinal endoscopy continues to advance, various methods to enhance mucosal imaging continue to be developed and applied. Besides the progression from fiber optic endoscopes to standard resolution video endoscopes to high-resolution video endoscopes, several other techniques and technologies for enhancing mucosal imaging have been introduced and used. The most widely studied method is dye-based chromoendoscopy. Dye-based chromoendoscopy involves application of dyes or contrast stains directly to the mucosa and is often untidy and time consuming. Potential alternatives to chromoendoscopy involve mucosal enhancing technologies such as Olympus narrow band imaging (NBI), PENTAX i-scan, and FUJI Fice. PENTAX i-scan is a software-driven technology that allows for per pixel modifications of sharpness, hue, and contrast to modify and enhance mucosal imaging. 

## 2. Background

**Figure 1:**
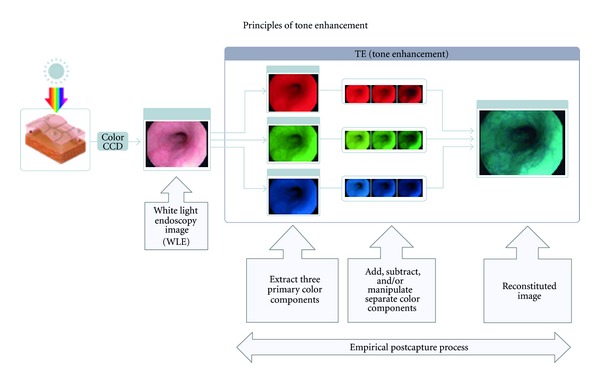
Principles of tone enhancement used by the postimage acquisition software in the endoscopic processor in i-scan technology. (Courtesy of Pentax Imaging, Pentax of America Montvale, NJ, USA).

i-scan uses postimage acquisition software with real time mapping technology embedded in the endoscopic processor (EPKi). The computer-controlled digital processing provides resolution of 1.25 megapixels per image, which allows for analysis and modification of the per pixel luminosity data. It does so by using various combinations of three software algorithms: surface enhancement (SE), contrast enhancement (CE), and tone enhancement (TE). Surface enhancement improves light/dark contrast, making dark areas appear darker and light areas appear lighter to better delineate edges and lesion borders. Contrast enhancement slightly suppresses red and green wavelength components of the white light image, while adding a minute blue hue to darker or more depressed areas of mucosa to allow for detailed observation of subtle mucosal irregularities. This helps define “peaks and valleys” within the mucosal surface, resulting in a more detailed topography of the mucosal surface. Tone enhancement analyzes all three components (red, blue, and green) of the white light image, and then dissects out and suppresses most of the dominant red, creating an image with an elevated blue/green contrast for detecting more subtle mucosal abnormalities (Figure 1). 

i-scan incorporates these three software algorithms into three distinct modes for the endoscopist: i-scan mode 1, i-scan mode 2, and i-scan mode 3. Each mode can be accessed or changed by a one button press on the endoscope. i-scan 1, designed as a surveillance mode uses SE and CE to provide more detailed topography of the mucosal surface and delineation of lesion edges without altering color. i-scan 2 and 3 also use SE and CE, but adds in TE to dissect out the dominant red and leave an elevated blue/green contrast (i-scan 2 darker contrast compared to i-scan 3). TE enhances vessel structures and minute mucosal structures to further pronounce margins of identified lesions. 

We report a series of 20 cases from a single academic tertiary care hospital where i-scan technology significantly impacted the management of patients in a wide variety of clinical scenarios. 

## 3. Methods

Consecutive cases were selected from our institution's endoscopy archive. Approval to review and report the selected cases was obtained from the University of Wisconsin Health Sciences Institutional Review Board. Informed consent was obtained from all participating patients. Data was collected on location of lesion (upper versus lower GI tract), diagnosis, i-scan mode, mucosal image detected, and impact on management. We compared and contrasted mucosal lesions detected with high-definition white light endoscopy (HD-WLE) versus i-scan image enhancement and the application on diagnostic sampling and therapy.

## 4. Results

i-scan image enhancement technology was used to diagnose and treat endoscopic findings and pathology in 13 cases involving the upper GI tract and 7 cases of the lower GI tract. Of the upper GI tract pathology, endoscopic image enhancement assisted in diagnosis, and/or therapy of 5 cases of Barrett's esophagus (BE) with dysplasia, 1 case each of T1 esophageal adenocarcinoma, HSV ulcerative esophagitis, gastric MALT lymphoma, distal gastric antral intestinal metaplasia with dysplasia, a flat duodenal adenoma with high grade dysplasia, and 3 cases of duodenal follicular lymphoma. Lower GI tract findings and pathology detected by i-scan imaging included 2 right-sided serrated polyps >1 cm, 1 case each of flat tubular adenoma, T1NO rectal adenocarcinoma, T1NO anal squamous cell cancer, solitary rectal ulcer, and radiation proctitis (Table 1).

### 4.1. Upper GI Tract

The following clinical scenarios illustrate the diverse utility of i-scan in detecting several different types of upper GI tract mucosal abnormalities including neoplasms with dysplasia and those with malignant and infectious etiologies. 

#### 4.1.1. Esophageal Dysplasia

**Figure 2:**
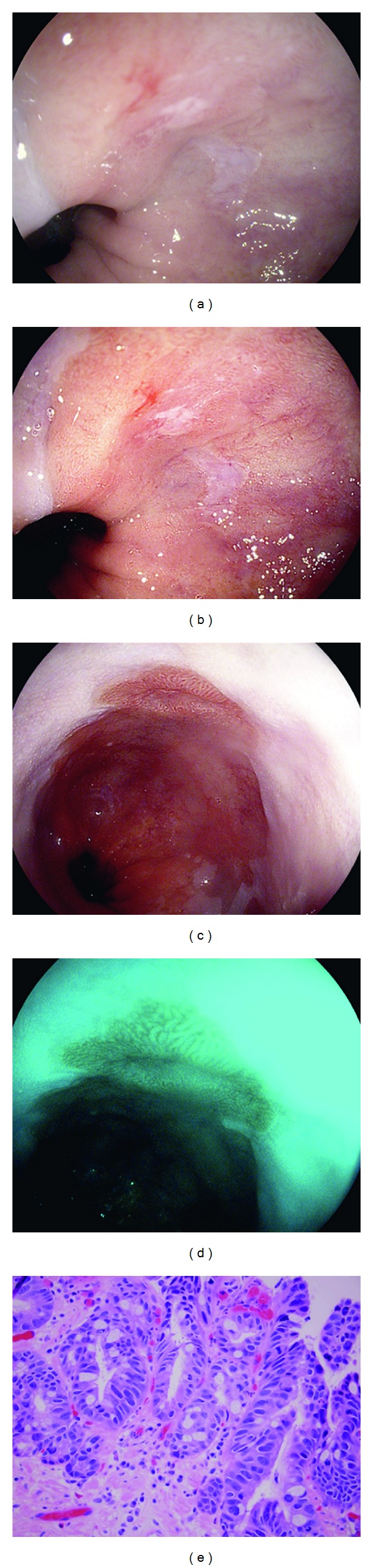
Barrett's esophagus with nodularity and high-grade dysplasia (HGD) visualized with HD-WLE (a), i-scan mode 1 (b) and (c), and i-scan mode 3 (d). Histopathology of EMR specimen showing Barrett's esophagus with HGD, H&E stain, and 400x magnification (e).

A 67-year-old man with long-standing GERD was referred for evaluation of short segment Barrett's esophagus with high-grade dysplasia. An endoscopic ultrasound (EUS) was performed and confirmed there was no invading submucosal lesions. EGD with HD-WLE demonstrated 2 cm of Barrett's mucosa (Figure 2(a)). With i-scan activated an erythematous area of nodularity within the Barrett's was better defined (Figures 2(b), 2(c), and 2(d)). This area was then removed in an overlapping fashion using the endoscopic mucosal resection (EMR) band ligation device. Histopathology of the EMR specimen demonstrated high-grade dysplasia (HGD) within the central nodule and surrounding low-grade dysplasia (Figure 2(e)). He subsequently returned two months later and underwent endoscopic radiofrequency ablation therapy (RFA). Followup surveillance endoscopy performed every 3 months for one year duration has not demonstrated recurrence of dysplasia or Barrett's esophagus. 

A 71-year-old man with previously diagnosed Barrett's esophagus with low-grade dysplasia (LGD) was referred for possible RFA of the Barrett's. EGD was performed and revealed a 5 cm segment of Barrett's esophagus. WLE revealed a possible small nodule within the segment of Barrett's. i-scan modes 1 and 2 were turned on which clearly defined a nodule within the Barrett's. This allowed for targeted resection via EMR. Pathology from the EMR revealed HGD. He returned for EGD 2 months later and no further nodules were present. The remainder of his Barrett's was successfully treated with endoscopic RFA.

Three additional cases of Barrett's with esophageal nodularity and HGD were seen. The first was a 66-year-old female with known 4 cm, long segment Barrett's with HGD. Repeat EGD with i-scan modes 1–3 highlighted nodular areas in her distal esophagus which aided in targeted EMR of those areas. Next, a 78-year-old man with Barrett's with HGD underwent EGD with planned RFA. Upon initial evaluation with WLE a 4 cm tongue of Barrett's was noted. Visualization using i-scan modes 1 and 2 highlighted 2 focal nodules that were removed with targeted EMR prior to RFA. Pathology from the nodule resections showed intestinal metaplasia with very high-grade dysplasia and submucosal telangiectasia. Subsequent EGD's have shown reduction in the extent of his Barrett's. Lastly, a 54-year-old man with known Barrett's with high-grade dysplasia and normal EUS had a repeat EGD which showed irregular nodular mucosa in scattered islands. i-scan modes 2 and 3 were used to accentuate the abnormal areas to allow for optimal EMR. 

#### 4.1.2. Esophageal Cancer

**Figure 3:**
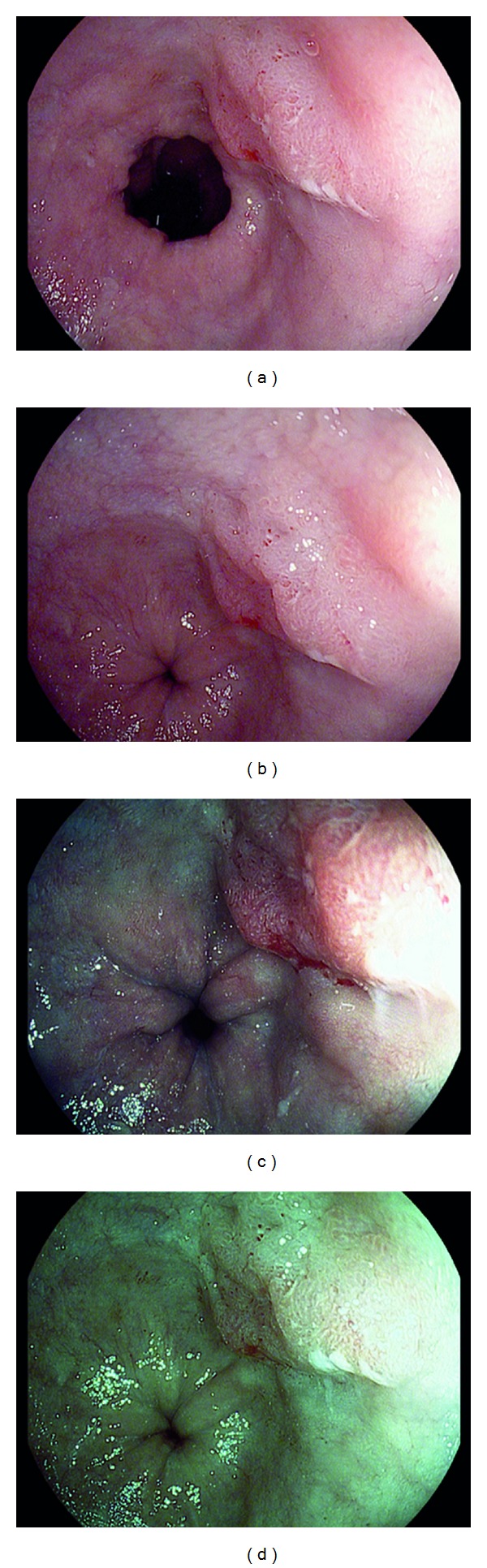
Raised nodule of adenocarcinoma within a segment of Barrett's esophagus as seen with HD-WLE (a), i-scan mode 1 (b), i-scan mode 2 (c), and i-scan mode 3 (d).

A 73-year-old man with a 10 year history of GERD was referred for EGD and EUS for long segment Barrett's esophagus with nodularity. On EGD the patient had 7 cm segment of Barrett's esophagus, with a single are of nodularity as seen on HD-WLE endoscopy image (Figure 3(a)). i-scan Modes 1, 2, and 3 (Figures 3(b), 3(c), and 3(d)) demonstrated the surface enhancement and margins of the nodule, thus targeting EMR. The nodule was removed with EMR technique and completely excised. Pathology demonstrated Adenocarcinoma confined to the muscularis propria layer with the resection margins free of any cancer or high-grade dysplasia. The patient then followedup 2 months later to begin treatment with RFA for the remaining Barrett's.

#### 4.1.3. Infectious Esophagitis

An 80-year-old man with history of orthotopic heart transplant presented with progressive dysphagia and odynophagia. Initial EGD demonstrated with WLE alone and biopsy sampling suggested mild, superficial candida esophagitis. He was treated appropriately with a 14-day course of fluconazole. However, his dysphagia and odynophagia persisted. After 10 days of antifungal therapy as an inpatient, a followup EGD was requested. Areas of mucosal irregularity and mild ulceration were visualized with WLE. Further examination with i-scan modes 1–3 showed severe distal esophagitis, with deep ulcerations, which allowed for guided biopsies to be taken for viral culture and staining. Pathology revealed positive immunostaining for HSV I and II. He was started on treatment dose acyclovir and transitioned to valacyclovir prophylaxis with no recurrence of his esophagitis to date and marked improvement in symptoms.

#### 4.1.4. Gastric Neoplasia

**Figure 4:**
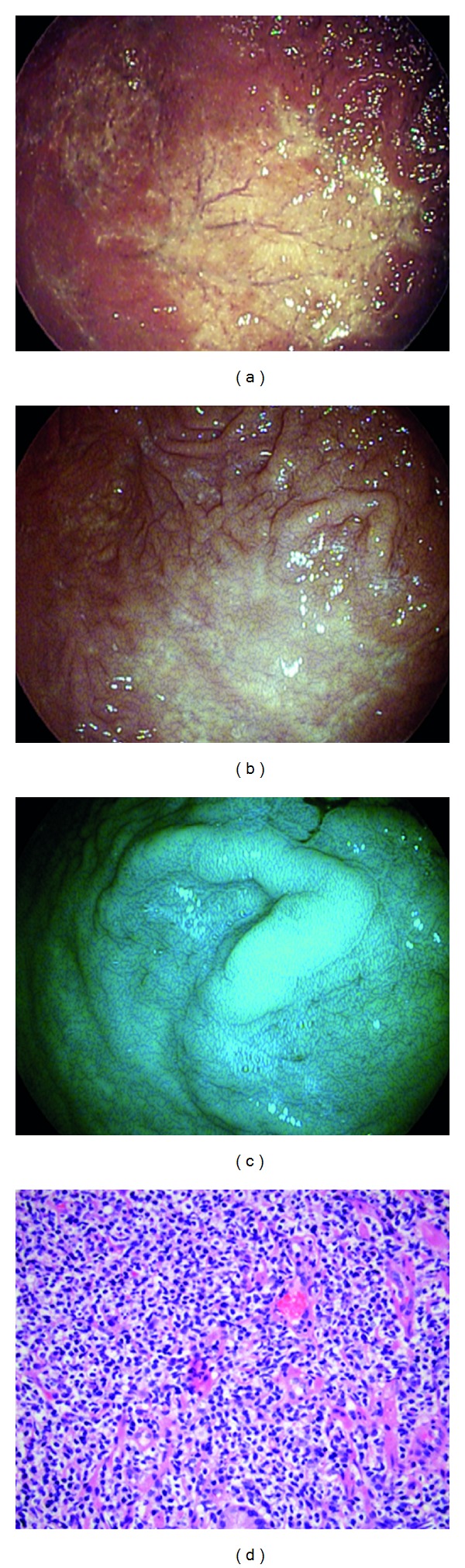
Gastric mucosa associated lymphoid tissue (MALT) lymphoma visualized with HD-WLE (a), i-scan mode 1 (b), and i-scan mode 3 (c). Histopathology showing MALT lymphoma, H&E stain, and 400x magnification (d).

A 64-year-old man was referred for epigastric pain, nausea, bloating, and dyspepsia. He denied constitutional symptoms of weight loss, fevers, chills, or rigors. Physical examination findings and index laboratory testing were within normal limits. A two-week trial of PPI prescribed by his primary care physician did not relieve his symptoms. He was referred for an EGD. On EGD, in the body and fundus of the stomach, WLE demonstrated pale, atrophic-appearing mucosa that could be consistent with gastritis or gastropathy (Figure 4(a)). i-scan was activated and demonstrated a patch of pale yellow mucosa with surrounding nodular, edematous gastric folds (Figures 4(b) and 4(c)). The nodular folds were apparent and prominent using i-scan imaging and allowed for targeted biopsies to be taken. The histopathology demonstrated low-grade mucosa associated lymphoid tissue (MALT) lymphoma (Figure 4(d)). He underwent a two-week course of therapy for H. pylori eradication. Subsequently he also had a limited course of chemotherapy. Surveillance endoscopy using i-scan was initially performed at 3 month and then 6 month intervals over the following 2 years. Additional biopsies have found no recurrence of the MALT lymphoma.

 A 77-year-old female with history of previous gastric antral intestinal metaplasia with tubulovillous adenoma presented for followup EGD. Initially, WLE showed friable, nodular, and heterogeneous mucosa along the greater curvature of the stomach. i-scan modes 1–3 showed more pronounced involvement of the antrum in an irregular circumferential manner with mucosa that appeared somewhat fibrotic. Biopsies of the abnormal area showed chronic active gastritis with areas of intestinal metaplasia and dysplasia. The patient was then referred for surgical evaluation and is awaiting a subtotal gastrectomy.

#### 4.1.5. Small Intestinal Neoplasia

A 60-year-old woman was referred for EGD for symptoms of atypical, noncardiac chest pain. She also had been experiencing profound weakness without any other specific symptoms. On EGD, she was found to have a normal esophagus and stomach. Incidentally, she had an abnormal appearing polyp in the second portion of the duodenum. She was referred for possible ampullectomy. Upon repeat EGD, there was evidence of not only a large, irregular appearing periampullary polyp, but also multiple small satellite lesions. i-scan modes 1 and 2 were turned on and revealed more widespread distinct lesions. Directed biopsies of these lesions revealed grade 1 follicular lymphoma. The patient was referred to hematology. Staging CT of her chest, abdomen, and pelvis, as well as a bone marrow biopsy, revealed no other evidence of lymphoma. She has not required treatment and has been followed closely with observation and serial EGDs and CT scans.

 A 52-year-old man with history of alcohol abuse and past pulmonary embolism in the setting of positive lupus anticoagulant underwent EGD for evaluation of dyspepsia. EGD showed erosive esophagitis without Barrett's and a normal stomach. Inspection of the duodenum showed an area of mucosal irregularity with WLE and further visualization with i-scan modes 1–3 showed a sessile polyp that was removed and found to be an adenoma. Follow-up EGD showed a postpolypectomy scar and some areas of nodularity in the duodenum but biopsies obtained were normal.

 Two additional cases of follicular lymphoma highlighted by i-scan were discovered. First, a 72-year-old man with abdominal pain and a known pancreas head mass was evaluated with EGD and EUS for further diagnosis of the mass. EGD with WLE showed mild gastritis and a hiatal hernia with nodular mucosa in the second portion of the duodenum. i-scan modes 1 and 2 showed additional focal areas of nodularity that upon biopsy revealed grade 1-2 follicular lymphoma. EUS biopsy of the pancreas mass also showed lymphoma. The patient followed up locally for treatment of his lymphoma. Second, a 60-year-old female underwent interval surveillance EGD to monitor her known focal low-grade follicular lymphoma (staging CT and bone marrow biopsy were negative for involvement). Using i-scan modes 1 and 2 slight regression of the burden of lymphoma was seen. The patient has active hematology followup and surveillance with EGD and CT.

### 4.2. Lower GI Tract

The following collection of clinical scenarios represent i-scan's varied utility in detecting lower GI tract pathology. The cases range from detection of colonic polyps to that of anorectal malignancies and nonmalignant anorectal pathology.

#### 4.2.1. Colonic Polyps

A 66-year-old asymptomatic, average risk woman was referred for a screening colonoscopy. In the ascending colon there was a large sessile polyp visualized under HD-WLE. i-scan mode 1 more clearly visualized the borders of the polyp. The polyp was lifted with injection of 3 mL of saline and was then removed in piecemeal fashion with hot snare polypectomy. i-scan allowed for improved visualization to ensure the polyp was completely removed. The area was then tattooed for future surveillance. Histopathology revealed a serrated polyp. Surveillance colonoscopy 6 months later revealed no residual polyp tissue.

**Figure 5:**
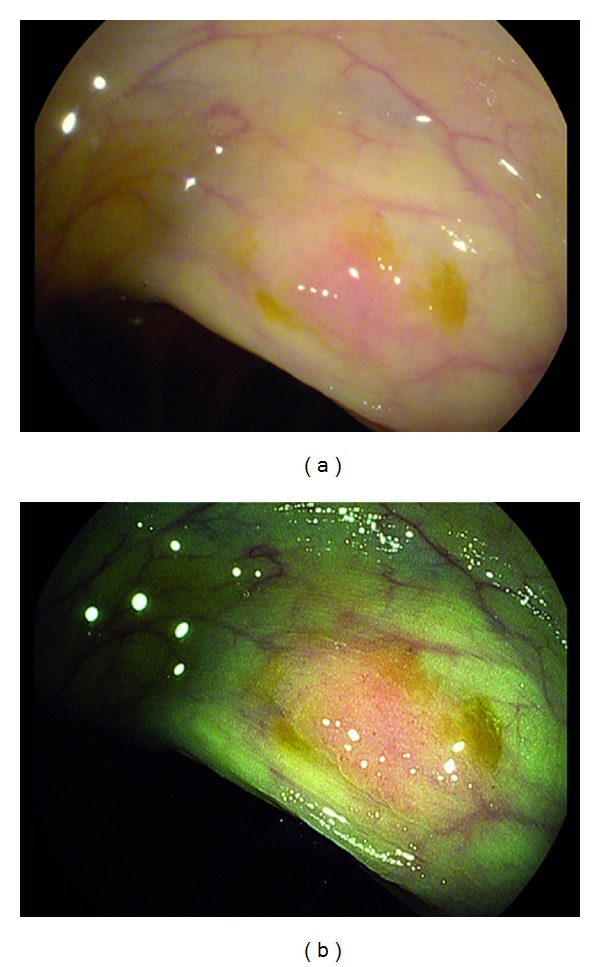
Sessile serrated polyp visualized under HD-WLE (a) and with i-scan mode 2 (b).

A 45-year-old man with no family history of colorectal cancer was referred for colonoscopy due to a change in bowel pattern. A minimally erythematous flat mucosal abnormality was noted in the ascending colon with WLE (Figure 5(a)). Further characterization with i-scan mode 2 showed a 7 mm polyp that was removed with hot snare (Figure 5(b)). Pathology revealed a sessile serrated polyp. He will have a repeat colonoscopy in 5 years.

A 64-year-old average risk man underwent routine colonoscopy for colorectal cancer screening. Near the ileocecal junction a fold appeared slightly prominent on WLE. Upon visualization with i-scan modes 1 and 2 a 2 cm sessile polyp was highlighted. Attempts to lift the polyp via saline injection for safe polypectomy were unsuccessful. The polyp was then biopsied and tattooed. An additional 6 mm sigmoid polyp was found and removed. Pathology showed tubular adenoma in both polyps. The patient underwent laparoscopic right hemicolectomy for the cecal adenoma and no higher grade lesions were discovered.

#### 4.2.2. Anorectal Malignancy

A 69-year-old man was referred for routine surveillance colonoscopy. He had a colonoscopy 5 years prior with a single tubular adenoma. Surveillance colonoscopy was normal throughout, including a retroflexed view in the rectum. Upon withdrawal of the scope, there was an area of mucosal irregularity noted at the dentate line. i-scan mode 1 was activated and revealed a small erythematous, friable, ulcerated nodular lesion in the distal anorectum in the posterior wall. This area was targeted for biopsy and the histopathology revealed squamous cell cancer of the anal canal. The patient had a rectal ultrasound which revealed a T1N0 lesion. He was referred to oncology and started on a combined chemotherapy and radiation regimen.

**Figure 6:**
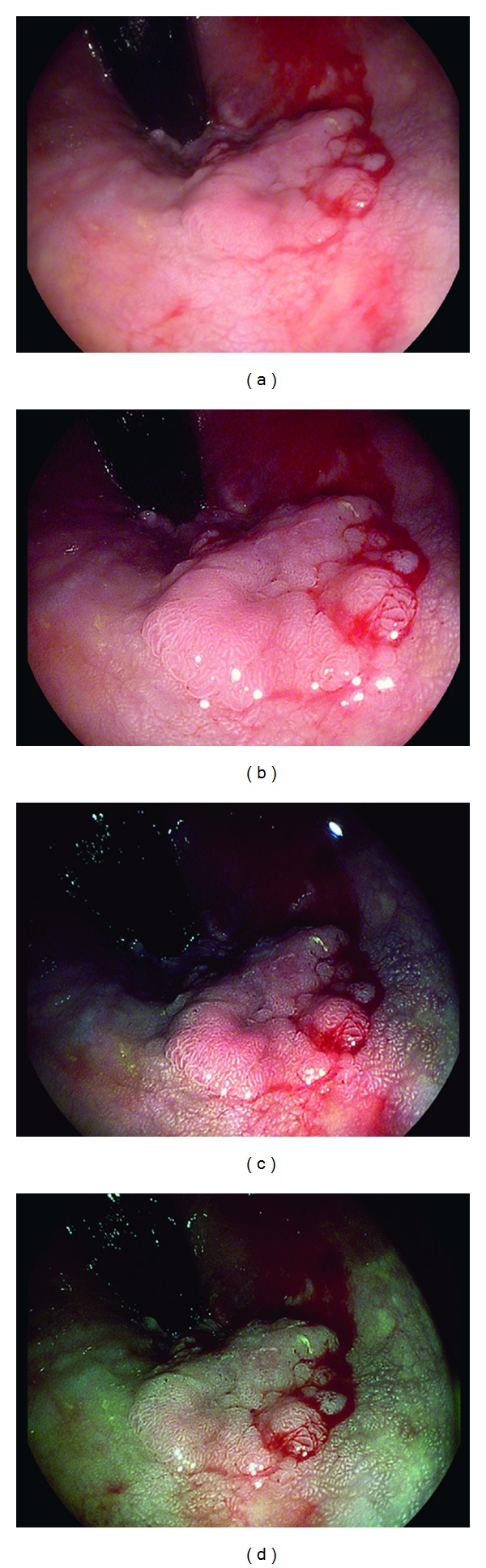
Large rectal polyp with focus of adenocarcinoma, visualized with HD-WLE (a), i-scan mode 1 (b), i-scan mode 2 (c), and i-scan mode 3 (d).

A 50-year-old asymptomatic, average risk woman underwent a screening virtual-CT colonography. An 8 mm polyp was seen in the sigmoid colon. She was referred for same day colonoscopy with polypectomy and the 8 mm polyp was removed. Upon retroflexion in the rectum, another 2 cm sessile polyp was seen (Figure 6(a)). i-scan modes 1, 2, and 3 were turned on and more clearly visualized the extent and borders of this large polyp and defined the lesion edges (Figures 6(b), 6(c), and 6(d)). The polyp was injected with saline and was removed completely in a piecemeal fashion using i-scan images to assess for completeness of resection. Pathology revealed tubulovillous adenoma and infiltrating well-differentiated adenocarcinoma extending to the cauterized margin. The patient was referred to colorectal surgery and underwent a transanal excision. Final surgical pathology revealed no residual carcinoma.

#### 4.2.3. Nonmalignant Anorectal Disease

An 81-year-old man with a history of prostate cancer treated with radiation was referred for a colonoscopy for evaluation of hematochezia. His screening colonoscopy five years earlier was normal. A colonoscopy was performed and revealed no polyps, tumors, or evidence of inflammatory bowel disease or ischemic colitis. In the distal rectum, there was the appearance of radiation proctitis seen on initial WLE which suggested the degree of proctitis was mild, encompassing approximately 25% of the surface area. However, i-scan mode 1 was turned on and revealed extensive radiation proctitis involving approximately 60% of the lumen surface area in the anterolateral wall in a semicircumferential fashion. This allowed for better targeting of therapy with argon plasma coagulation (APC). His symptoms of hematochezia improved. Two months later, he returned for one more follow-up flexible sigmoidoscopy with additional APC therapy of the radiation proctitis, which had significantly improved since the first colonoscopy. After followup one year later, he has had no further hematochezia.

Finally, a 64-year-old female with history of diarrhea and rectal bleeding was evaluated with colonoscopy. The colon was normal outside of some scattered diverticula and a couple of diminutive polyps. Further evaluation of her rectum with i-scan modes 1 and 2 showed a 6 mm solitary rectal ulcer. Biopsy of the ulcer edge showed benign squamous and columnar mucosa with focal neutrophilic infiltrates.

## 5. Discussion

There is an increasing demand for improving mucosal imaging as a means to identify pathology and target diagnostic sampling and guide endoscopic therapy. This has resulted in the development of technology-based methods for enhancing mucosal imaging. Digital filter technologies, such as i-scan, are becoming one of the more common and practical technologies to achieve this goal. We have presented a series of cases that illustrates the applicability of this technology across a broad spectrum of clinical scenarios.

Digital filter-based technologies like i-scan were developed out of the concept of dye-based chromoendoscopy. Dye-based chromoendoscopy involves manually applying dye or contrast directly to the mucosa to better delineate normal from pathologic mucosa. It can be done in either an untargeted fashion across the entire mucosal surface, or in a targeted fashion at specific lesions. Dye-based chromoendoscopy leads to improved visualization of mucosal irregularities and has been studied in various situations ranging from screening colonoscopy to ulcerative colitis and Barrett's surveillance [1–3]. However, while it still useful in some situations, dye-based chromoendoscopy can be cumbersome and increase procedure times. Also, following initially promising studies on dye-based chromoendoscopy, no further studies have been conducted and the data remains descriptive [4, 5]. 

There are several optical and digital filter techniques that have been developed to enhance mucosal imaging without manually applying contrast to the mucosa. Narrow band imaging (NBI) uses optical light filters in front of the light source to narrow the bandwidth of the projected light. This produces a colored image on the screen with enhanced contrast. NBI has not been shown to improve adenoma detection rates [6], but has been shown to improve discrimination of hyperplastic from adenomatous polyps [7]. When compared to standard WLE, NBI improves diagnostic yield in detecting dysplasia in Barrett's esophagus by detecting more dysplasia from fewer, more targeted biopsies [8].

As opposed to using filters at the light source as in NBI, newer proprietary technologies such as Fuji Intelligent Chromoendoscopy (FICE) (Fuji) and i-scan (Pentax) use postimage acquisition software to modify the images seen. FICE has been shown to be as good as dye chromoendoscopy in detecting colonic adenomas [9, 10]. FICE is also superior to conventional WLE, but equal to dye chromoendoscopy, in differentiating neoplastic from nonneoplastic colon polyps [11]. FICE can diagnose Barrett's esophagus with the ability to clearly demarcate between Barrett's mucosa and gastric mucosa [12]. FICE has also been shown to be equivalent to conventional chromoendoscopy with acetic acid in detecting neoplasia in Barrett's esophagus [13]. i-scan has been shown to significantly increase the detection of small colon polyps sized 5 mm or less [14]. i-scan in combination with high-definition colonoscopy has also been shown to improve overall adenoma detection compared to standard white light colonoscopy alone [15]. In the esophagus, i-scan can help identify reflux-associated mucosal breaks, but it has not been extensively studied in the setting of Barrett's esophagus [16]. 

We have illustrated a variety of cases where i-scan technology can be clinically useful and directly impact management of patients. i-scan was able to better detect and enhance mucosal abnormalities throughout the gastrointestinal tract compared to HD-WLE. Lesions that were highlighted ranged from Barrett's esophagus and gastric MALT lymphoma to duodenal follicular lymphoma and rectal adenocarcinoma. In general i-scan allowed for more precise delineation of lesion borders to allow for more complete mucosal resection and/or biopsy. In several of the cases there were significant changes in outcomes that may have been missed if WLE was used alone. Specifically, in the case involving the periampullary follicular lymphoma i-scan was able to further delineate the extent of a duodenal lymphoma, preventing unnecessary ampullectomy with its potential morbidities.

This case series adds to the growing body of literature supporting the clinical utility of enhanced mucosal imaging. The main limitations of this series are that the experiences were limited to a single center and a few endoscopists experienced in the use of i-scan. The validity of i-scan to more readily detect mucosal abnormalities compared to current WLE will need to be addressed in more detail by prospective multicenter studies. Currently there are only single institution studies reported in the literature [14, 15, 17, 18].

i-scan technology shows promise as a useful modality in a wide array of clinical scenarios throughout the gastrointestinal tract to better detect and treat mucosal abnormalities. It can provide more detailed topography of the mucosal surface and delineate lesion edges by enhancing vessel and minute mucosal structures. Utilizing i-scan endoscopic image enhancement may directly impact the management of patients.

## Figures and Tables

**Table 1:** Cases in which i-scan imaging highlighted mucosal abnormalities not as clearly seen with white light endoscopy and subsequently affected management.

Case no.	Diagnosis	i-scan mode	Mucosal image	Impact on management
Esophagus				
(1)	BE with HGD	1, 3	Nodule of HGD	Targeted EMR
(2)	BE with LGD	1, 2	Nodule of LGD	Targeted EMR
(3)	BE with HGD	1, 2, 3	Nodularity with HGD	Targeted EMR
(4)	BE with HGD	1, 2	Nodularity with HGD	Targeted EMR
(5)	BE with HGD	1, 2, 3	Nodularity with HGD	Targeted EMR
(6)	Esophageal cancer	1, 2, 3	Accentuated abnormal tissue	Targeted EMR
(7)	HSV esophagitis	1, 2, 3	Deep ulcerations	Targeted biopsy

Stomach				
(8)	Gastric MALT lymphoma	1, 3	Gastric folds mucosal abnormality	Targeted EMR
(9)	CAG with intestinal metaplasia and dysplasia	1, 2, 3	Highlighted gastric thickening and nodularity	Subtotal gastrectomy

Small intestine				
(10)	Periampullary follicular lymphoma	1, 2	Identified extent of involvement	Prevented unnecessary ampullectomy
(11)	Duodenal adenoma with dysplasia	1, 2, 3	Highlighted flat polyp margins	Complete EMR
(12)	Grade 1-2 submucosal follicular lymphoma	1, 2	Highlighted lymphoid appearance	Targeted EMR and prevention of surgical excision
(13)	Low-grade follicular lymphoma	1, 2	Highlighted nodular area	Targeted biopsy

Colon and rectum				
(14)	Serrated adenoma	1, 2	Margins of polyp	Polyp detection and polypectomy
(15)	Serrated adenoma	2	Accentuated borders of right-sided polyp	Complete polypectomy
(16)	Tubular adenoma	1, 2	Detailed border of polyp on fold	R hemicolectomy
(17)	Anal SCCa T1N0	1, 2	Identified mucosal abnormality in anal canal	Targeted Bx
(18)	Rectal adenocarcinoma T1N0	1, 2, 3	Identified borders of flat “depressed” rectal polyp	Targeted complete polypectomy
(19)	Radiation proctitis	1	Identified extent of involvement	Allowed for more diffuse APC
(20)	Solitary rectal ulcer	1, 2	Accentuated subtle ulcer	Targeted Bx

Abbreviations: BE: Barrett's esophagus, LGD: low-grade dysplasia, HGD: high-grade dysplasia, EMR: endoscopic mucosal resection, HSV: herpes simplex virus, MALT: mucosa-associated lymphoid tissue, CAG: chronic active gastritis, SCCa: squamous cell carcinoma, and APC:  argon plasma coagulation.
